# Tricuspid Valve Infective Endocarditis in a Chronic Haemodialysis Patient with a Hickman Catheter: A Case Report

**DOI:** 10.3390/pathogens14060539

**Published:** 2025-05-28

**Authors:** Dalila Šačić, Saddam Shawamri, Ivana Jovanović, Marija Boričić-Kostić, Boris Jegorović, Miloš Mijalković, Kristina Filić, Stefan Juričić, Vidna Karadžić-Ristanović, Danka Bjelić, Selena Gajić, Marko Baralić

**Affiliations:** 1 Clinic for Cardiology, University Clinical Center of Serbia, 11000 Belgrade, Serbia; 2 Faculty of Medicine, University of Belgrade, 11000 Belgrade, Serbia; 3 Clinic for Infectious and Tropical Diseases “Prof. dr Kosta Todorovic”, University Clinical Center of Serbia, 11000 Belgrade, Serbia; 4 Polyclinic Kardio Medika, 18000 Niš, Serbia; 5 Clinic for Nephrology, University Clinical Center of Serbia, 11000 Belgrade, Serbia

**Keywords:** hemodialysis, Hickman catheter, infective endocarditis, *Enterococcus* spp.

## Abstract

Infective endocarditis (IE) of the tricuspid and pulmonary valve accounts for 5 to 10% of all IE cases and, compared with left-sided IE, is often associated with intravenous (i.v.) drug use, presence of intracardiac devices, and central venous catheters (CVCs), including permanent—Hickman catheter (HC). We report a case of a 71-year-old female patient on a chronic hemodialysis (HD) program who had developed IE. Her first symptoms were fever and malaise. Transthoracic echocardiography (TTE) and transesophageal echocardiography (TEE) examinations were performed, revealing vegetations on the tip of HC and the anterior and posterior leaflets of the tricuspid valve (TV). Three blood culture bottles were positive for *Enterococcus* spp. The HC was replaced with a new CVC to continue HD. After a six-week antibiotic treatment, most clinical symptoms were resolved, and there was a decrease in vegetation size with normalization of inflammatory markers and negative follow-up blood cultures. After this initial improvement in the patient’s condition, the clinical course was complicated by the development of *Citrobacter koseri* bacteremia and sepsis. Despite adequate antibiotic therapy, the condition progressed to septic shock, which was soon followed by a fatal outcome. IE treatment in HD patients requires long-term broad-spectrum antibiotic therapy, and also, in patients without arteriovenous fistula (AVF), the CVC should be replaced after each HD during IE and sepsis treatment to minimize the patient’s exposure to a foreign body that is susceptible to bacterial colonization. A colonized foreign body is a focus for sustained and spreading infection, and its presence prevents adequate antibiotic treatment until the focus of infection is removed.

## 1. Introduction

Infective endocarditis (IE) is a global health concern characterized by infections affecting the native or prosthetic heart valves, the mural endocardium, a septal defect, or an indwelling cardiac device [1]. IE has an annual incidence of 3–10/100,000 of the population. The epidemiology has gradually changed over the years, with healthcare-associated IE now accounting for 25–30% of contemporary cohorts due to the greater use of intravenous lines and intracardiac devices [2].

*Enterococci* are gram-positive, facultative anaerobic commensal organisms of the gastrointestinal tract and known uropathogens. *Enterococcus* spp. has become a leading cause of healthcare-associated infections, especially in immunocompromised patients. These bacteria are responsible for a wide range of infections, including urinary tract infections, bacteraemia, endocarditis, intra-abdominal infections, and surgical site infections [3,4].

*Enterococcus*-related IE generally affects an elderly and fragile population, with a high mortality rate. Diagnosis of *Enterococcus*-related IE is challenging, especially in anuric patients or those without known diverticulosis, due to its often-subacute course, with nonspecific constitutional symptoms and chronic anemia, which is difficult to interpret in an elderly and frequently immunosuppressed population with a large number of comorbidities. Management is also challenging due to *Enterococcus* antimicrobial resistance [5].

Right-sided infective endocarditis (RSIE) accounts for 5 to 10% of all IE cases and, compared with left-sided IE, is often associated with intravenous drug use (IVDU), intracardiac devices, and central venous catheters (CVCs), including the Hickman catheter (HC) [6,7]. Despite diagnostic and therapeutic advances, the mortality of IE is still high, and the long-term prognosis remains uncertain [8].

We present the case of a patient on a chronic hemodialysis (HD) program with a permanent HC and tricuspid valve (TV) IE. Due to the complexity of treatment, the patient’s CVC became the focus of a sustained infection, even as that patient had no other option for HD access than the use of such a CVC.

## 2. Case Presentation

We report the case of a 71-year-old female patient with a history of end-stage kidney disease (ESKD)—in this case, secondary to polycystic kidney disease—who had been treated with HD for 12 years. Due to thrombosis of an arteriovenous fistula (AVF), complicated by cellulitis, HD was continued through an HC placed in the right jugular vein. The patient was anuric, with good compliance. The kidney replacement therapy (KRT) was competent, with adequate HD and a Kt/V greater than 1.2. There were no data for the presence of complicated renal cysts in the patient. Besides ESKD, personal history was positive for hypertension (HTN) and stroke, followed by a brain aneurysm operation. A left mastectomy was performed due to breast cancer. She also had dementia.

The present illness started with a fever of up to 38 °C and malaise, without other specific symptoms. Due to the ongoing COVID-19 pandemic, the patient was examined by an infectious disease (ID) specialist in the triage center, which ruled out Severe Acute Respiratory Syndrome Coronavirus 2 (SARS-CoV-2) infection as a cause of the patient’s illness. The patient was admitted to the Clinic for Nephrology, University Clinical Center of Serbia (UCCS) for further treatment.

A month before her current febrile state, she was treated surgically in the orthopedic clinic for an open distal femur fracture, with the placement of an external fixator. Antibiotic prophylaxis was administered.

On admission, the patient was awake, disoriented, dysarthric, febrile, immobile, and pale. Vital signs were normal. An HC was present on the right side of the upper chest with no signs of inflammation of the surrounding skin. Heart sounds were clear, with a systolic murmur heard best in the fourth and fifth right intercostal spaces. Auscultation of the lungs revealed diminished breath sounds bilaterally, though more on the right side. Abdominal ultrasound showed enlarged polycystic kidneys with one cyst of cloudy content in the left kidney. We could not take a urine sample for urine culture as the patient was anuric. The rest of the examination was unremarkable. Due to the patient’s altered conscious state, a neurologist indicated a CT scan of the endocranium, which revealed a chronic subdural hematoma. Due to high fever and HC, we suspected *Staphylococcus* bacteriemia, so, immediately after admission, intravenous (i.v.) vancomycin therapy was started, with an initial dose of 1 g i.v., then 750 mg i.v. every other day after HD, with continued dosing according to the drug level in the blood. The external fixator of the femur was removed. The wound from the removed fixator site was without signs of inflammation, and *Staphylococcus coagulase-negative* was isolated from the wound swab.

**Figure 1:**
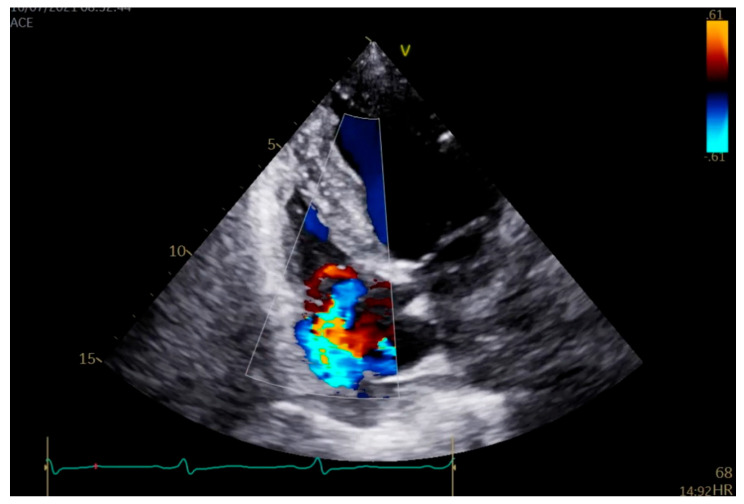
Transthoracic image (four chamber view). Color Doppler showing massive tricuspid regurgitation caused by the infective endocarditis of the tricuspid valve.

**Figure 2:**
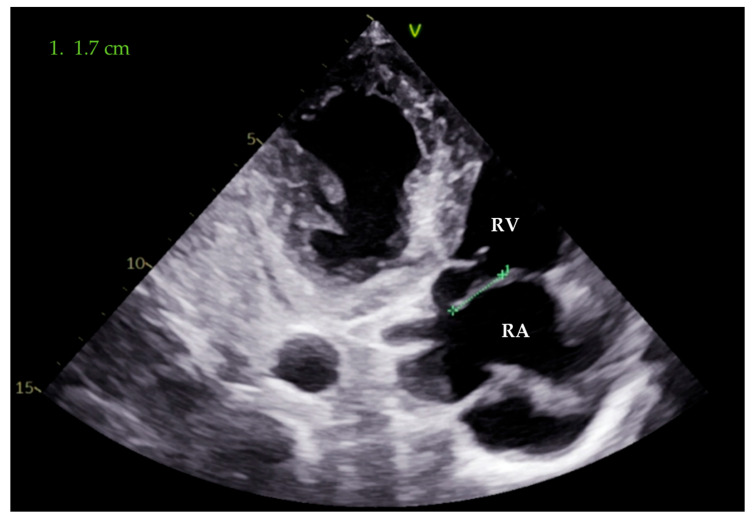
Transthoracic image (apical four chamber view, right ventricle focused). The length of the vegetation attached to the anterior leaflet of the tricuspid valve is 1.7 cm (green line). RA—right atrium; RV—right ventricle.

**Figure 3:**
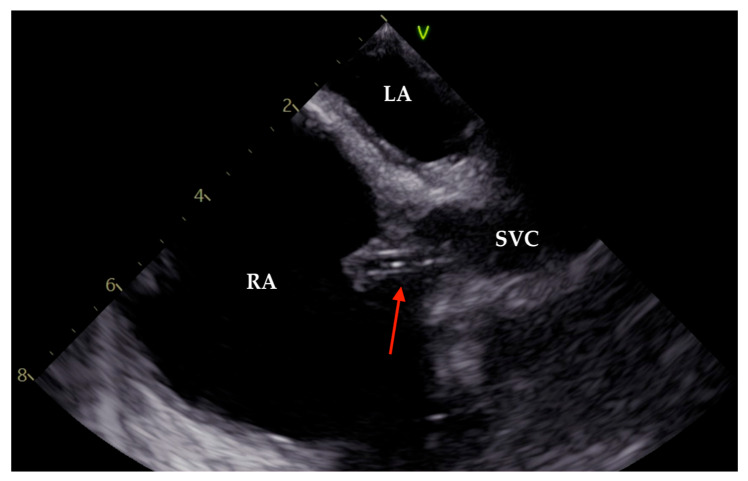
Transesophageal image (93°). Tip of the Hickmann catheter (red arrow), located in the superior vena cava and entering the right atrium, covered with the vegetation mass. LA—left atrium; SVC—superior vena cava; RA—right atrium.

**Figure 4:**
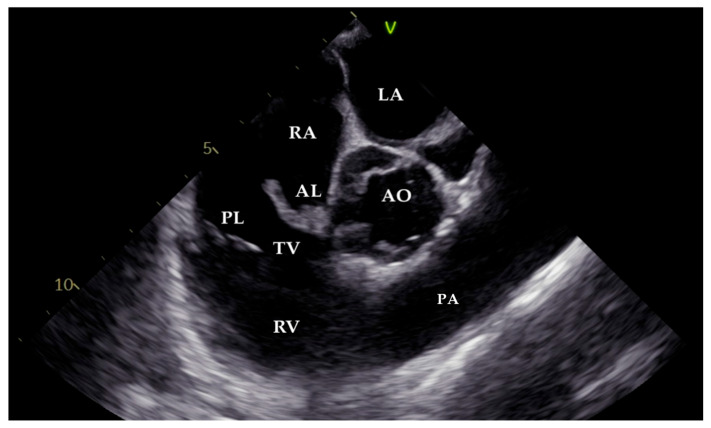
Transesophageal image (70°). Vegetation masses covering the anterior leaflet of the tricuspid valve. LA—left atrium; RA—right atrium; RV—right ventricle; AO—aorta; TV—tricuspid valve; AL—anterior leaflet; PL—posterior leaflet; PA—pulmonary artery.

**Figure 5:**
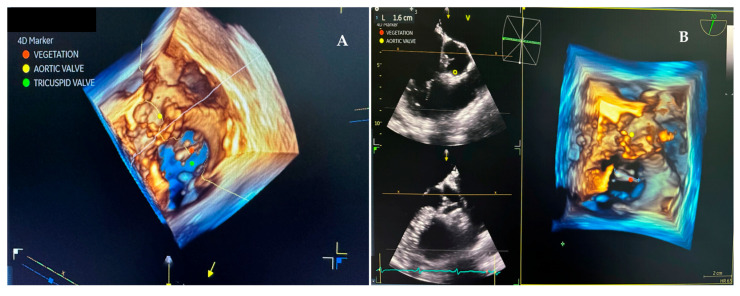
Transesophageal image (70°) RT3D acquisition. (**A**) Yellow dot—aortic valve; green dot—tricuspid valve; red dot—vegetation mass. (**B**) Yellow dot—aortic valve; red dot—vegetation measured 1.6 cm in length.

As the patient remained febrile during the next few days despite antibiotic therapy, and because there was worsening of an existing heart murmur, a transthoracic echocardiogram (TTE) examination was performed, which showed vegetations on the anterior leaflet of the tricuspid valve (TV), the largest measuring 1.7 cm in length. These findings were associated with massive tricuspid regurgitation (TR). Indirectly estimated right ventricular systolic pressure (RVSP) was 45 mmHg (Figure 1 and Figure 2). After that, transesophageal echocardiography (TEE) was performed, confirming the presence of vegetations on the anterior TV leaflet. The severity of TR remained unchanged, but, this time, the distal tip of the HC at the entrance to the right atrium was clearly visible and covered in echogenic masses suggestive of vegetations (Figure 3, Figure 4 and Figure 5).

Immediately after IE was confirmed on TEE, the HC was removed, and the catheter tip was sent for microbiological analysis. The HC tip culture remained sterile. The patient was then transferred to the clinic for cardiology at the UCCS for further treatment. On admission, a physical examination revealed a systolic murmur over Erb’s point and diminished breath sounds in the basal parts of both lungs, with rare late inspiratory crackles. Blood pressure (BP) was 150/85 mmHg. An electrocardiogram (ECG) showed sinus rhythm with a left bundle branch block pattern. The results of laboratory analyses performed upon admission are shown in Table 1.

Because continued KRT was necessary, a new central CVC was placed in the patient’s right subclavian vein 48 h after the removal of the HC. As the peripheral blood culture sampled during the ID examination became positive for *Enterococcus* spp., the antibiotic therapy was switched from vancomycin to a combination of ampicillin (2 g i.v. every 12 h) and linezolid (600 mg i.v. every 12 h), according to the antibiogram of the isolated bacteria. Two new peripheral blood culture samples were taken. After five days of incubation, both new blood culture bottles grew the same pan-sensitive *Enterococcus* spp. again. Blood cultures taken from HC lines were sterile. The patient was treated with the antibiotic combination for the next six weeks, resulting in improved clinical and laboratory parameters, and follow-up blood cultures were negative. Repeated echocardiographic examinations showed a gradual decrease in the size of existing vegetations.

After normalization of inflammatory parameters, the patient was transferred back to the clinic for nephrology for further management, due to the need for permanent access for HD. Due to CVC malfunction, which developed over the following days, a new dialysis CVC was placed in the left jugular vein, and HD was continued without complications. The patient developed diarrhea with a fever of up to 38.5 °C and a significant rise in inflammatory markers (C-reactive protein was 188.1 mg/L), followed by the development of sepsis and deterioration of the level of consciousness. Meropenem was prescribed empirically before blood culture results were available. Soon afterward, the patient became hypotensive with the need for vasopressor support. Repeated blood cultures grew *Citrobacter koseri* sensitive to third-generation cephalosporins, carbapenems, aminoglycosides, and fluoroquinolones. However, despite the antibiotic and supportive treatment, the patient’s condition deteriorated, and two weeks later, the patient died. 

## 3. Discussion

In the past, IE affected younger adults with rheumatic heart valve disease, but it now also affects older patients with or without previously known valve disease, who more often develop IE as the result of healthcare-associated procedures [9]. Additionally, new predisposing factors have appeared, such as degenerative valvular disease, IVDU, HD, and various invasive diagnostic and therapeutic procedures. These have all led to a rising trend in the incidence of IE [10]. The development of infectious endocarditis requires the presence of bacteria or fungi in the blood and an intracardiac surface on which these microorganisms can attach [11]. Although bacteria are not normally present in the blood, any skin and mucosal injury can cause transitory bacteremia. Certain dental, surgical, and other medical procedures, such as intravenous catheter placement, can introduce bacteria into the bloodstream [12,13]. In our case, the primary suspected source of IE was the presence of a right atrial indwelling HC as a permanent access for HD, but blood cultures taken from the HC lines and the tip culture of the removed HC were sterile. Possible reasons for sterile cultures include antibiotic therapy initiated before cultures were collected for analysis, or another possible focus of infection that led to the development of IE. Our patient had kidney cysts, which, even in anuric patients, can be a focus for bacterial colonization and further dissemination of bacteria that can colonize the valves. The third possible focus of bacteriemia and IE in our patient was previous femur surgery and wounds of the external femur fixator. The surgery was performed with antibiotic prophylaxis, and the wounds were regularly dressed without signs of inflammation. After the external fixator was removed, Staphylococcus coagulase-negative, a conditionally pathogenic bacterium that is part of the physiological flora of the skin, was isolated from the wound swab.

A variety of complications, both infectious and non-infectious, can arise with the use of HC, but infectious complications are the most common problem associated with HC use. Such infections include catheter colonization, exit site infection, tunnel infection, catheter-related bacteremia or fungemia, septic thrombophlebitis, and infusate-related bloodstream infection [14]. Our patient’s additional risk factor for IE was immunodepression due to long-term HD treatment. Patients with chronic kidney disease (CKD) have disturbances of immune function involving both innate and adaptive systems. These result in both immunodepression, which increases susceptibility to infection, and immunoactivation, leading to a chronic inflammatory state. Dialysis treatment may further aggravate aspects of this [15].

It is estimated that gram-positive bacteria account for approximately 80% of cases of native-valve infective endocarditis (NVIE) worldwide. In most cases of NVIE, bacteria are identified [16]. *Staphylococcus* and *Streptococcus* are the most prevalent microorganisms, accounting for 25.9–44.1% and 19.0–33.8% of cases, respectively. Most staphylococcus are *S. aureus* (18.6% to 33.4%). Viridans group *Streptococci* are the predominant species following *S. aureus* (9.1–17.8%). *Enterococcus* is the third most common etiology (3.2–15.8%) [17], and is gaining relevance, especially among healthcare-associated cases. Patients with *Enterococci* IE are older and have more comorbidities than patients with other types of IE [18], according to our case, as our patient was >70 years old with many comorbidities and more than one possible focus of bacteriemia and IE.

Symptoms in our patient were acute onset high-grade fever, malaise, disorientation, dysarthria, and newly diagnosed heart murmur, which, along with the risk factor of the presence of HC, indicated a high suspicion of the development of IE. The diagnosis of IE is based on clinical suspicion supported by consistent microbiological data, hemoculture, imaging, TTE, TEE, and documentation of IE-related cardiac lesions. Evidence of involvement of cardiac valves (native or prosthetic) or prosthetic intracardiac material is a primary diagnostic criterion of IE. Echocardiography is the first-line diagnostic imaging technique [19]. Our patient’s diagnosis was established based on two major Duke criteria: positive blood cultures and echocardiographically confirmed vegetation.

Treatment of IE should be started promptly, in hospital conditions, with i.v. antimicrobial drugs and cardiac surgery when it is indicated. There are three main reasons to undergo surgery in the setting of IE: heart failure, uncontrolled infection, and prevention of septic embolization (in particular, to the CNS) [19]. Empiric antimicrobial therapy in native valve endocarditis targets the three most common pathogens: *Staphylococcus*, *Streptococcus*, and *Enterococci* [20]. Native valve endocarditis treatment typically requires 2 to 6 weeks of antibiotics [19]. Treatment of *Enterococcal* IE has long been recognized as an important clinical challenge. *Enterococci* are difficult to treat as they have several defense mechanisms that allow resistance to a wide range of antibiotics. The frequent lack of bactericidal activity of traditional agents (penicillin or ampicillin) and the increased reports of high-level resistances to aminoglycosides, in parallel with the production of bacterial biofilms over prosthetic devices, have led to a much higher rate of relapse (7–10%) compared with other etiologies. These relapses can occur still several months after the end of the antimicrobial therapy, generating continuous uncertainty for the clinician and the need for a prolonged follow-up. Treating *Enterococcal* IE requires combining two antimicrobials to achieve synergy for at least 4 to 6 weeks [5]. Due to high fever and HC, we suspected *Staphylococcus* bacteriemia in our patient, so, immediately after admission, began i.v. vancomycin treatment. After we received the blood culture sample result, we changed antibiotic therapy according to the antibiogram and continued antibiotic treatment for the next six weeks, which resulted in improved clinical and laboratory parameters, a negative follow-up blood culture sample, and a decrease in the size of existing vegetation on echocardiographic examinations. Compared with left-sided IE, there are relatively little data on surgery for RSIE and few clinical practice guidelines [21]. Surgical management in our patient was initially postponed due to an increased risk of peri- and postoperative complications that were due to multiple comorbidities and were later abandoned because of the improvement in the patient’s condition, absence of fever, normalization of inflammatory markers and reduction in vegetations size. This followed existing literature, which states that surgery is necessary if antibiotics fail in the case of RSIE, whereas in left-sided IE, early surgery is often required [22]. In the case report of Tomoaia et al., the heart team decided for valve replacement in a patient with double valve IE caused by *Pseudomonas aeruginosa*, despite improved inflammatory symptoms and negative control blood cultures, and the patient survived [23]. Bentata et al. have also reported a case of severe TV IE caused by *Pseudomonas aeruginosa* in a patient on a chronic HD program with a tunneled CVC as a permanent HD access. Their patient improved after CVC removal and antibiotic treatment [24]. By searching the literature, we did not find cases similar to ours, such as TV IE caused by *Enterococcus* spp. in a chronic HD patient with an HC, with which we could compare our results.

After the initial improvement in the general condition of our patient, with the ultrasound-proven reduction of vegetation and the normalization of inflammation parameters, their general condition worsened again, with the development of sepsis and then death. The exact source of the infection was not determined because, due to the sudden deterioration of the patient’s condition and technical difficulties, it was impossible to perform all the necessary diagnostics, including a colonoscopy and a control ultrasound of the heart. Possible sources were complicated renal cysts, colonic diverticulitis, wound at the site of a previously placed external femoral fixator, or partial remnant of previous TV vegetation.

Factors associated with an increased risk of IE in in-hospital mortality include IE complications (severe valvular insufficiency, vegetations, valvular perforation or aneurysm, new partial dehiscence of prosthetic valve, formation of abscesses, pseudoaneurysm or intracardiac fistula), the development of heart failure, septic shock, local uncontrolled infection, as well as the lack of surgical treatment [25,26].

The limitation of this study was that we did not perform the control echocardiography after the rapid deterioration of the patient’s general condition due to technical difficulties in the nephrology department, where the patient was transferred from the cardiology department for further treatment.

## 4. Conclusions

Fever in HD patients with CVC should prompt early suspicion of RSIE. Starting an appropriate antibiotic treatment as soon as the first symptoms occur and possible signs of IE are assumed, along with the timely removal of infected CVC or other focuses of infection, are crucial for treatment success. A colonized foreign body is a focus for sustained and spreading infection, and its presence prevents adequate treatment with antibiotics until the infective focus is removed.

Managing IE in HD patients requires long-term, often broad-spectrum antibiotic regimens and a multidisciplinary approach. In HD patients, an immunocompromised group, the members of normal bacterial flora may act as an opportunistic pathogen and cause severe infection.

In HD patients without AVF, the CVC should be replaced after each HD during IE and sepsis treatment to minimize the patient’s exposure to a foreign body susceptible to bacterial colonization.

Long-term follow-up of patients with IE is essential because, despite the current clinical improvement, relapse or new bloodstream infections may complicate the course of treatment.

## Figures and Tables

**Table 1:** Laboratory findings at admission.

Laboratory Values	Hospital Admission	Reference Range
CRP, mg/L	**112**	0.0–5.0
Pct, ng/mL	**0.45**	<0.05
Ferritin, ug/L	**1704**	13.0–150
Fibrinogen, g/L	3.5	2.0–4.0
ESR, mm/h	**48**	<30
Leucocytes, 10^9^/L	5.0	3.4–9.7
Erythrocytes, 10^12^/L	**3.71**	4.34–5.72
Hemoglobin, g/L	**104**	122–157
Platelets, 10^9^/L	153	150–450
Glucose, mmol/L	4.4	3.9–6.1
Serum urea, mmol/L	**8.4**	2.5–7.5
Serum creatinine, μmol/L	**506**	45–84
Uric acid, μmol/L	370	150–400
Sodium, mmol/L	137	135–148
Potassium, mmol/L	3.6	3.5–5.1
LDH, U/L	339	220–460
iPTH, ng/L	21	15–65

CRP—C-reactive protein, Pct—procalcitonin, ESR—erythrocytes sedimentation rate, LDH—lactate dehydrogenase, iPTH—intact parathyreoid hormone. Abnormal values are bolded.

## Data Availability

The data presented in this study are available on request from the corresponding author due to privacy restrictions.

## References

[1] Li M., Kim J.B., Sastry B.K.S., Chen M. (2024). Infective endocarditis. Lancet.

[2] Rajani R., Klein J.L. (2020). Infective endocarditis: A contemporary update. Clin. Med..

[3] Sangiorgio G., Calvo M., Migliorisi G., Campanile F., Stefani S. (2024). The Impact of *Enterococcus spp.* in the Immunocompromised Host: A Comprehensive Review. Pathogens.

[4] Codelia-Anjum A., Lerner L.B., Elterman D., Zorn K.C., Bhojani N., Chughtai B. (2023). *Enterococcal* Urinary Tract Infections: A Review of the Pathogenicity, Epidemiology, and Treatment. Antibiotics.

[5] Herrera-Hidalgo L., Fernández-Rubio B., Luque-Márquez R., López-Cortés L.E., Gil-Navarro M.V., de Alarcón A. (2023). Treatment of *Enterococcus faecalis* Infective Endocarditis: A Continuing Challenge. Antibiotics.

[6] Akinosoglou K., Apostolakis E., Marangos M., Pasvol G. (2013). Native valve right sided infective endocarditis. Eur. J. Intern. Med..

[7] Yanagawa B., Bahji A., Lamba W., Tan D.H., Cheema A., Syed I., Verma S. (2018). Endocarditis in the setting of IDU: Multidisciplinary management. Curr. Opin. Cardiol..

[8] Scheggi V., Merilli I., Marcucci R., Del Pace S., Olivotto I., Zoppetti N., Ceschia N., Andrei V., Alterini B., Stefàno P.L. (2021). Predictors of mortality and adverse events in patients with infective endocarditis: A retrospective real world study in a surgical centre. BMC Cardiovasc. Disord..

[9] Sheppard N.M. (2022). Infective endocarditis. Diagn. Histopathol..

[10] Pant S., Patel N., Deshmukh A., Golwala H., Patel N., Badheka A., Hirsch G.A., Mehta J.L. (2015). Trends in infective endocarditis incidence, microbiology, and valve replacement in the United States from 2000 to 2011. J. Am. Coll. Cardiol..

[11] Pierce D., Calkins B.C., Thornton K. (2012). Infectious endocarditis: Diagnosis and treatment. Am. Fam. Physician.

[12] Minasyan H. (2019). Sepsis: Mechanisms of bacterial injury to the patient. Scand. J. Trauma. Resusc. Emerg. Med..

[13] Wilson W., Taubert K.A., Gewitz M., Lockhart P.B., Baddour L.M., Levison M., Bolger A., Cabell C.H., Takahashi M., Baltimore R.S. (2007). Prevention of infective endocarditis: Guidelines from the American Heart Association: A guideline from the American Heart Association Rheumatic Fever, Endocarditis, and Kawasaki Disease Committee, Council on Cardiovascular Disease in the Young, and the Council on Clinical Cardiology, Council on Cardiovascular Surgery and Anesthesia, and the Quality of Care and Outcomes Research Interdisciplinary Working Group. Circulation.

[14] Yip C., Rotstein C. (1998). Hickman catheter-related infections in patients with cancer. Int. J. Antimicrob. Agents.

[15] Ball J.B., Gopaluni S., Mathavakkannan S., Brian M., Farrington K. (2010). An immunocompromised dialysis patient with skin and bone lesions. NDT Plus.

[16] Nappi F. (2024). Native Infective Endocarditis: A State-of-the-Art-Review. Microorganisms.

[17] Miao H., Zhang Y., Zhang Y., Zhang J. (2024). Update on the epidemiology, diagnosis, and management of infective endocarditis: A review. Trends Cardiovasc. Med..

[18] Pericás J.M., Zboromyrska Y., Cervera C., Castañeda X., Almela M., Garcia-de-la-Maria C., Mestres C., Falces C., Quintana E., Ninot S. (2015). *Enterococcal* endocarditis revisited. Future Microbiol..

[19] Delgado V., Ajmone-Marsan N., de Waha S., Bonaros N., Brida M., Burri H., Caselli S., Doenst T., Ederhy S., Erba P.A. (2023). 2023 ESC Guidelines for the management of endocarditis. Eur. Heart J..

[20] Wang A., Athan E., Pappas P.A., Fowler V.G., Olaison L., Paré C., Almirante B., Muñoz P., Rizzi M., Naber C. (2007). Contemporary clinical profile and outcome of prosthetic valve endocarditis. JAMA.

[21] Habib G., Lancellotti P., Antunes M.J., Bongiorni M.G., Casalta J.-P., Del Zotti F., Dulgheru R., El Khoury G., Erba P.A., Iung B. (2015). 2015 ESC Guidelines for the management of infective endocarditis: The Task Force for the Management of Infective Endocarditis of the European Society of Cardiology (ESC). Endorsed by: European Association for Cardio-Thoracic Surgery (EACTS), the European Association of Nuclear Medicine (EANM). Eur. Heart J..

[22] Lin T.I., Huang Y.F., Liu P.Y., Chou C.A., Chen Y.S., Chen Y.Y., Hsieh K.S., Chen Y.S. (2016). *Pseudomonas aeruginosa* infective endocarditis in patients who do not use intravenous drugs: Analysis of risk factors and treatment outcomes. J. Microbiol. Immunol. Infect..

[23] Tomoaia R., Oprea A., Sandu I., Danu V., Pop D., Zdrenghea D., Dădârlat-Pop A., Minciună I.A., Chețan I.M., Hada N.C. (2022). A Rare Case of Successfully Treated Double Valve Infective Endocarditis Caused by *Pseudomonas aeruginosa*. Int. J. Mol. Sci..

[24] Bentata Y., Haddiya I., Ismailli N., Benzirare A., Elmahi O., Azzouzi A. (2013). Severe tricuspid valve endocarditis related to tunneled catheters in chronic hemodialysis patients: When should the catheter be removed?. Arab J. Nephrol. Transplant..

[25] Marques A., Cruz I., Caldeira D., Alegria S., Gomes A.C., Broa A.L., João I., Pereira H. (2020). Risk Factors for In-Hospital Mortality in Infective Endocarditis. Arq. Bras. Cardiol..

[26] Leblebicioglu H., Yilmaz H., Tasova Y., Alp E., Saba R., Caylan R., Bakir M., Akbulut A., Arda B., Esen S. (2006). Characteristics and analysis of risk factors for mortality in infective endocarditis. Eur. J. Epidemiol..

